# Supplementary figures and images for: Endurance exercise with reduced muscle glycogen content influences substrate utilization and attenuates acute mTORC1- and autophagic signaling in human type I and type II muscle fibers
Source: Skelet Muscle. 2025 Mar 25;15:9. doi: 10.1186/s13395-025-00377-3 (PMC11934587; doi:10.1186/s13395-025-00377-3)

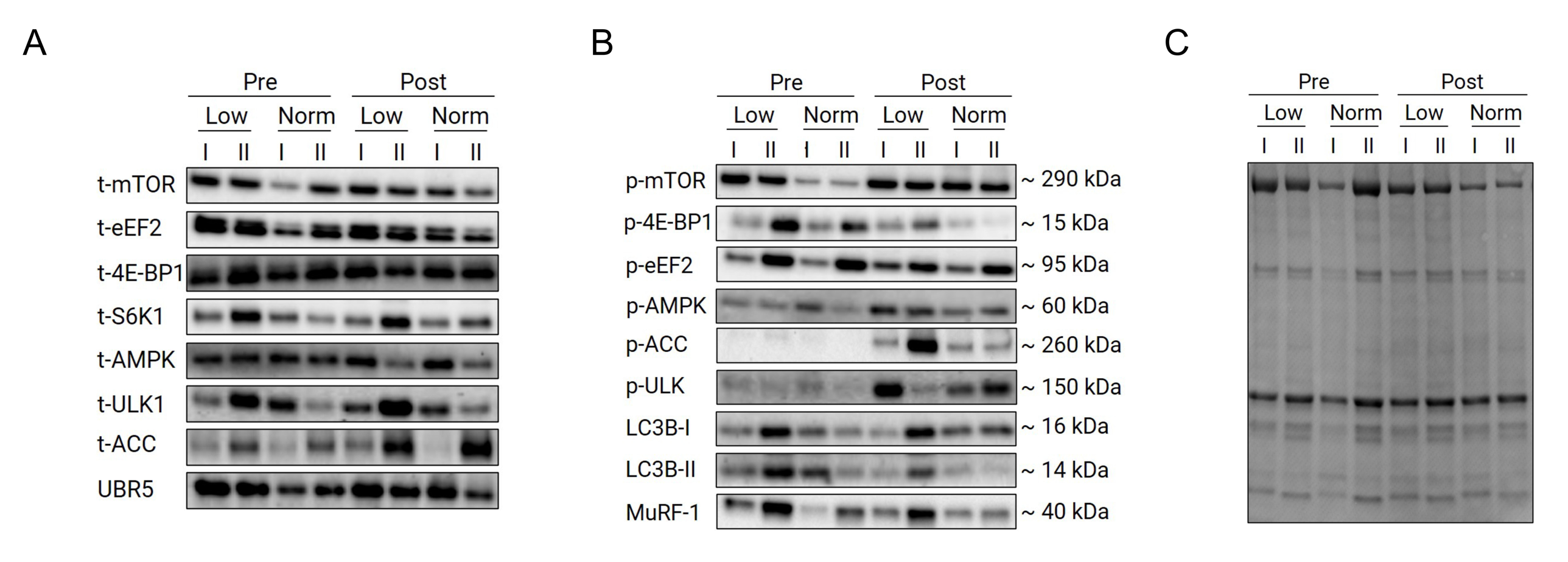

Supplement: Supplementary file 4 — Supplementary Material 4: Supplementary figure 4. Representative immunoblots of total protein content (A), phosphorylated protein (B) and total protein stain (MemCode) (C). [file 13395_2025_377_MOESM4_ESM.jpg]
